# Early-life influenza A (H1N1) infection independently programs brain connectivity, HPA AXIS and tissue-specific gene expression profiles

**DOI:** 10.1038/s41598-024-56601-5

**Published:** 2024-03-11

**Authors:** Myriam P. Merz, Snehaa V. Seal, Nathalie Grova, Sophie Mériaux, Pauline Guebels, Georgia Kanli, Elise Mommaerts, Nathalie Nicot, Tony Kaoma, Olivier Keunen, Petr V. Nazarov, Jonathan D. Turner

**Affiliations:** 1 Immune Endocrine and Epigenetics Research Group, Department of Infection and Immunity, Luxembourg Institute of Health (LIH), 29 Rue Henri Koch, 4354 Esch-Sur-Alzette, Luxembourg; 2 Faculty of Science, Technology and Medicine, University of Luxembourg, 2 Avenue de Université, L-4365 Esch-Sur-Alzette, Luxembourg; 3 Inserm U1256, NGERE, Nutrition-Génétique Et Exposition Aux Risques Environnementaux, Université de Lorraine, 54000 Nancy, France; 4 In Vivo Imaging Platform, Luxembourg Institute of Health, 1445 Strassen, Luxembourg; 5 Translational Radiomics, Department of Cancer Research, Luxembourg Institute of Health, 1526 Luxembourg, Luxembourg; 6 LuxGen Genome Center, Laboratoire National de Santé, Luxembourg Institute of Health, 3555 Dudelange, Luxembourg; 7 Bioinformatics Platform, Data Integration and Analysis Unit, Luxembourg Institute of Health, 1445 Strassen, Luxembourg; 8 Multiomics Data Science Research Group, Department of Cancer Research, Luxembourg Institute of Health, 1445 Strassen, Luxembourg; 9 Present Address: Central Biobank Charité, Berlin Institute of Health at Charité–Universitätsmedizin Berlin, Berlin, Germany

**Keywords:** Early life adversity, Infection, HPA axis, Stress, Corticosterone, Gluconeogenesis, Multi-organ programming, H1N1, Influenza, MRI, Neuroimmunology, Translational immunology, Epigenetic memory, Behavioural methods, Imaging, Stress and resilience

## Abstract

Early-life adversity covers a range of physical, social and environmental stressors. Acute viral infections in early life are a major source of such adversity and have been associated with a broad spectrum of later-life effects outside the immune system or “off-target”. These include an altered hypothalamus–pituitary–adrenal (HPA) axis and metabolic reactions. Here, we used a murine post-natal day 14 (PND 14) Influenza A (H1N1) infection model and applied a semi-holistic approach including phenotypic measurements, gene expression arrays and diffusion neuroimaging techniques to investigate HPA axis dysregulation, energy metabolism and brain connectivity. By PND 56 the H1N1 infection had been resolved, and there was no residual gene expression signature of immune cell infiltration into the liver, adrenal gland or brain tissues examined nor of immune-related signalling. A resolved early-life H1N1 infection had sex-specific effects. We observed retarded growth of males and altered pre-stress (baseline) blood glucose and corticosterone levels at PND42 after the infection was resolved. Cerebral MRI scans identified reduced connectivity in the cortex, midbrain and cerebellum that were accompanied by tissue-specific gene expression signatures. Gene set enrichment analysis confirmed that these were tissue-specific changes with few common pathways. Early-life infection independently affected each of the systems and this was independent of HPA axis or immune perturbations.

## Introduction

Early life adversity (ELA) is used as a collective term to describe physiologically or psychologically stressful events or environments early in life, with significant adverse effects on long-term health trajectories. While there are no clearly defined age boundaries for this early life period, the “first 1000 days of life” (from conception to about 2 years of age) are commonly thought to encompass this period of vulnerability^1^. Although initially covering the direct effects of the early life period, this developmental origins of health and disease theory has been refined and it is thought that such exposure renders the individual more vulnerable to the deleterious stimuli and ultimately contribute to the onset of chronic non-communicable pathologies, such as diabetes, autoimmune and cardiovascular diseases, as well as mental disorders^2–4^. This is the “three-hit model” where the largely unchangeable genome is the “first hit”, the early life exposure is the “second hit”, and then the disease risk is crystallized when a third environmental hit is encountered later in life.

ELA comes in many forms, such as the exposure to environmental toxins, the lifestyle and social implications of a low socio-economic status (SES), the stressful environment of a dysfunctional household or the deeply embedded trauma of childhood abuse^5^. While several health outcomes in children (e.g. fetal growth restrictions, preterm birth, or fetal alcohol syndrome) are clearly re-traceable to specific, identifiable, adverse conditions or risk behaviors during pregnancy^6–8^, assessing the impact of diverse postnatal environmental and social exposures is far more diverse and harder to dissect as the adversity experienced is rarely due to a single event, but rather the sum of many diverse individual elements. This is particularly true for the most studied human model of postnatal adversity: the institutionalization-adoption paradigm^9^. Here, the adversity includes separation from the mother combined with the decreased care and human bonding, exposure to an increased microbial, infectious or antigenic load as well as possible malnourishment^10–12^. In this paradigm, these stimuli coalesce and are implicated in improper development of the HPA axis^13,14^, impaired brain maturation^15,16^, and an accelerated ageing of the immune system^17–21^.

Early-life infections (ELI) are an essential element of the overall ELA burden. ELI poses both an immediate risk from the infection itself, as well as long term consequences including altered development ^22,23^, increased risk of childhood asthma or allergies^24,25^ developement of type 1 diabetes^26^, as well as inducing changes in cytokine production and stress reactivity lifelong^27^. The relatively high permeability of the blood–brain barrier in early life means that high peripheral cytokine levels (e.g. interleukin 1β [IL-1β], IL-6 and tumour necrosis factor α [TNFα]) can cross the barrier and potentially cause long-term memory impairments. Increased exposure to cytomegalovirus (CMV) infection together with the higher overall risk of childhood infections due to the institutionalisation^21,28^ has been associated with accelerated immune ageing and immunosenescence^20,21,29^. One important observation was that the age of first infection is a crucial factor in the later life immune competence^30^ and may affect all other consequences of ELI as well.

As ELA and ELI are major risk factors involved in the pathophysiology of many stress-related disorders^2–4^, it was long assumed that both the autonomic nervous system and the hypothalamic–pituitary–adrenal (HPA) axis stress systems were central to the underlying mechanism^31,32^. Indeed, there is a large body of evidence connecting physical stress (e.g. malnutrition, infection) with a series of outcomes including HPA axis dysregulation, impaired immune activation and brain development^33–36^. However, data from our EpiPath cohort suggests that peripheral tissues are independently programmed by the early life environment and the long-term changes observed do not depend on an altered HPA axis^21,28,37,38^. This suggests that the mechanism linking ELA to the overall phenotype is somewhat more complicated than a simple dependence upon an altered HPA axis. Re-examining all the available literature led us to propose the “stem cell hypothesis of ELA pathogenesis”, where we suggested that ELA affects tissue-resident stem/progenitor cells in each tissue independently, and these then go on to differentiate into functionally impaired cells in lifelong^39^. The direct consequence of this is that we now think that ELA may have direct effects on many tissues, and the changes seen in HPA axis are one such outcome, rather than the supposed central controlling mechanism.

Here we have taken ELI, one element of the multipartite human ELA exposure. Using a murine ELI model we investigated the long-term impact of a such an infection later in life in tissues outside the immune systems that are known to be affected by ELA. We investigated the role of ELI on all aspects of the ELA phenotype including the development of the HPA axis function, hepatic glucose production, as well as brain connectivity and tissue-specific gene expression profiles.

## Results

**Figure 1:**
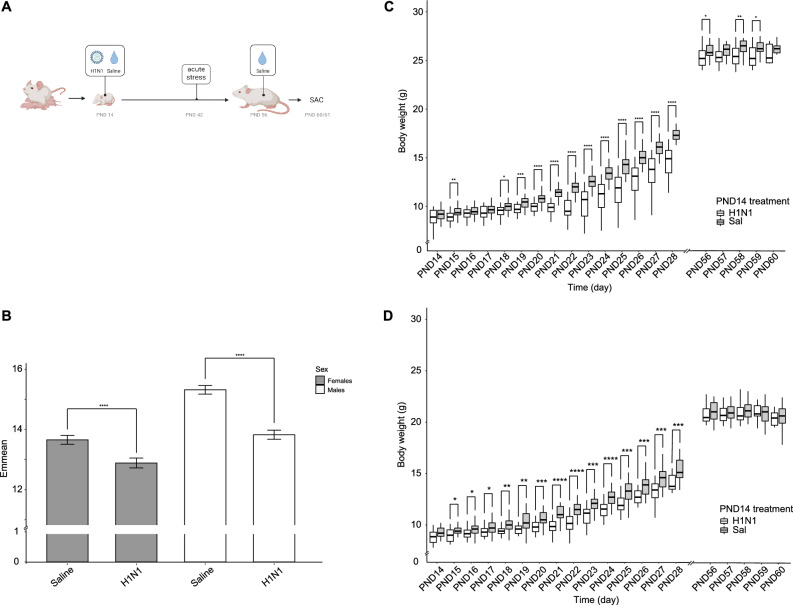
(**A**) Study outline: pups, born in-house, were subjected to early life infection (H1N1 or PBS) on post-natal day 14 (PND14). On PND42 all animals were subjected to an acute stress test in the form of restrained stress. Given ample time to recover from the stress test, mice were handled and mock-inoculated on PND56, approximately five days before being sacrificed on PND60/61. (**B**) Analysis of Covariance (ANCOVA) to evaluate the effect of two independent grouping variables (sex and treatment) on body weight, after adjustment for time. Estimated Marginal Means (emmean) of body weight shows significance (**** = p > 0.0001) between treatments (control = green; IAV = orange) with regards to sex. Error bars display SEM. C) Body weight development over time between different PND14 treatments for males and D) females. Boxplots display the distribution of data with the median and the inter quartile range (IQR), where individual animals are represented as points. Treatments are represented in colors (control = green; IAV = orange). P-values (*p > 0.05; **p > 0.01; ***p > 0.001; ****p > 0.0001) represent comparisons between treatments for each individual day.

The litters of 24 pregnant dams were randomly divided into two groups (12 each) and exposed to ELI treatment of either influenza-A-virus (IAV) dissolved in Phosphate-buffered saline (PBS) or PBS as control (Fig. 1A). We registered a slight litter-size effect for animals assigned to the control group (mean litter size 5.17 ± 2.82 SD) *vs*. the IAV treatment group (mean litter size 6.92 ± 1.83 SD). However, since litters were randomly assigned to a treatment before birth, this appears to be a coincidence. In total, we had a population of 153 mice exposed to ELI (control: n = 64, 32 females; IAV: n = 89, 46 females), of which 9 animals (4 females) did not survive the PND14 IAV infection.

### Early life H1N1 infection phenotype

Body weights were measured between PND14-PND28 and PND56-PND60 ± 1. As expected, body weight significantly increased in both groups (control and IAV infected mice) over time, regardless of sex (ANCOVA, Bonferroni corrected; PND14-PND60; p < 0.001 for control mice and p < 0.001 for IAV infected mice). Throughout the experiment, the controls always weighed more than the IAV-infected mice. Post-hoc analysis also showed a significant effect of sex on body weight starting around PND18-PND19. We performed a two-way ANCOVA examining the effects of sex and PND14 treatment on body weight over time. Regardless of time, both sex (F(1, 1823) = 303.8, p < 0.001) and PND14 treatment (F(1, 1823) = 223.0, p < 0.001) were statistically significant factors determining body weight. There was also a significant interaction between PND14 treatment and sex (F(1, 1823) = 22.1, p < 0.001), indicating that sex had a direct impact on PND14 treatment. Consequently, we used Estimated Marginal Means over the complete experimental period (PND14-60) for pairwise comparisons for both sex and PND14 treatment and applied a Bonferroni correction. We observed that the PND14 treatment affected the males (p < 0.001) much more strongly than the females (p < 0.001 ) (Fig. 1B). Based on these results, sexes were separately treated in all subsequent analyses.

Examining each time point independently, there was a trend that, prior to infection, pups in the PND14 IAV treatment group were lighter than those in the control group (8.79g ± 1.07 SD vs 9.27g ± 0.99SD; p > 0.05, Mann–Whitney U-Test ), which becomes significant at PND18 (p < 0.05, Mann–Whitney U-test). This initial difference at PND15-18 was most probably due to the differences in litter size rather than to a direct result of treatment, with a tendency towards fewer, bigger, pups in the control group. The weight difference reached a peak at PND22 (IAV vs control; p < 0.05, Mann–Whitney U-test), which period corresponds to 7–8 days after infection where IAV-sickness symptoms are the most acute (data not shown). While the IAV-infected females recovered their body weight by PND56-60 (p > 0.05; Mann–Whitney U-test), the males remained lighter than the controls (Fig. 1C,D; p < 0.05, Mann–Whitney U-test).

**Figure 2:**
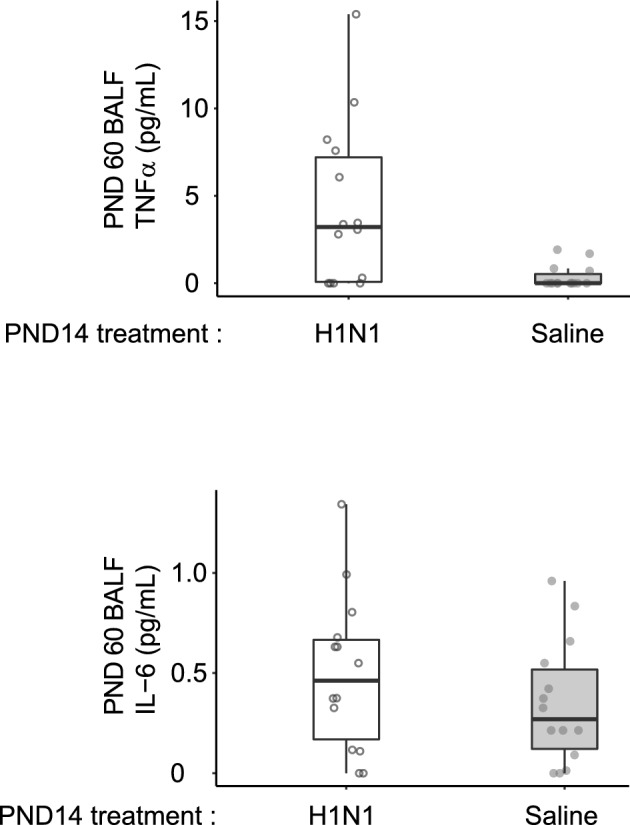
Cytokine levels at PND60. We measured IL-2, IL-6, IL-10, in the blood and IL-6, TNF-α and INF-γ in the bronchoalveolar lavage fluid (BALF). The only detectable cytokines were TNF-α and IL-6 (Fig. 2A and B respectively) Overall cytokine levels were very low (< 16 pg/mL, however differences were significant for TNF-α between IAV and control. We found no sex-specificity in cytokine levels. Boxplots display the distribution of data with the median and the inter quartile range (IQR), where individual animals are represented as points. Treatments are represented in colors (control = grey; IAV = unfilled). All animals only received PBS at PND56. Animals receiving IAV at PND56 were used as controls to ensure that assays worked and non-detection of BALF cytokines was not due to experimental error.

By PND60, all visible signs of IAV infection (abdominal breathing, hunched posture or decreased mobility) were resolved. IAV clearance from the lungs was confirmed by qPCR (Supplementary Data 1). There was no detectable viral amplification at PND60 within 40 PCR cycles in the PND14 IAV-infected animals. Cytokine levels were measured in blood and bronco alveolar lavage fluid (BALF). We did not find detectable levels of interleukin 2 (IL-2), IL-6 or IL-10 in the blood of randomly-tested animals (n = 10) confirming the resolution of the infection. Since IAV is a respiratory virus, we also measured IL-6, TNF-α and INF-γ levels in the BALF of randomly-selected animals (n = 28). Very low levels of IL-6 and TNF-α were measured in IAV infected animals whereas INF-γ was not detected in any of our samples. Although levels of TNF-α were overall low, they were higher in the IAV-infected mice (Mann–Whitney U test; p = 0.005, n = 28: IAV median = 3.22 pg/mL; control median = 0 pg/mL; Fig. 2A). Levels of IL-6 in the IA-infected (median = 0.46 pg/mL) animals were similar to those in the control mice (median = 0.27 pg/mL; Mann–Whitney U test; p = 0.333, n = 28; Fig. 2B). Furthermore, there was no significant sex difference for IL-6 (Mann–Whitney U test; p = 0.332, n = 28) or TNF-α (Mann–Whitney U test; p = 1.00, n = 28) in BALF.

### Acute stress glucose and corticosterone response

**Figure 3:**
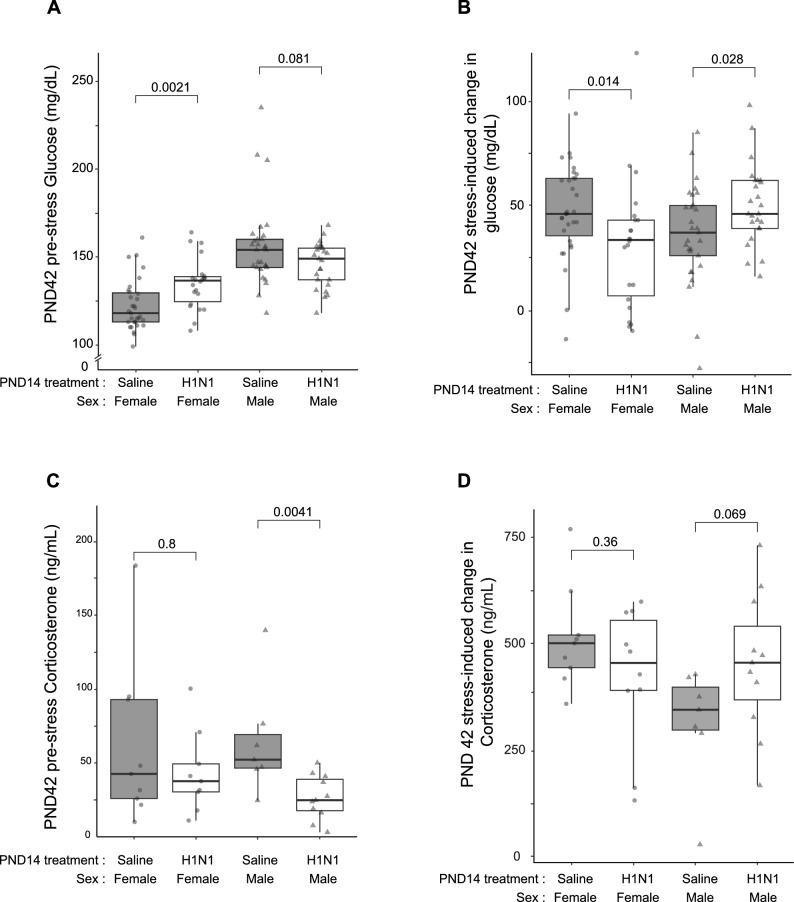
Changes in blood glucose and corticosterone in response to acute stress performed at PND42. (**A**) Pre-stress levels of blood glucose (mg/dL) for females and males on PND42, separated by PND14 treatment group. (**B**) Rise in glucose during stress test (pre- to post-restraint). Results visualized as median per PND14 treatment (color) and sex (line type) ± S.E.M. (**C**) Pre-stress plasma corticosterone (CORT) levels on PND42 (ng/mL) separated by PND14 treatment group. (**D**) Rise in CORT during the PND42 stress test (pre- to post-restraint). Results visualized as median per PND14 treatment group (color) and sex (point type) ± SEM. All boxplots display the distribution of data with the median and the inter quartile range (IQR). Whiskers show the minimum and maximum as 1.5*IQR. Individual animals are visualized by shapes.

The restraint-stress-induced glucose and corticosterone release was assessed on PND42. As we had already observed a sex-specific effect for body weight, we used a two-way ANOVA to evaluate the effect of sex on treatment, confirming that sex has the biggest effect on glucose levels (p < 0.001) and an interaction effect with treatment (sex*PND14 treatment; p < 0.001). Consequently, we analyzed female and male glucose levels separately. We observed a clear effect of treatment on glucose levels prior to stress onset in the females (Mann–Whitney U test; p = 0.01; n = 53; Fig. 3A) between the control group (median = 118, IQR = 113–129.5, n = 31) and the IAV-infected mice (median = 136.5, IQR = 124.5–138.8, n = 22) with approximately 15% higher levels in median glucose. Interestingly, in males, we found pre-stress glucose levels to be slightly lower in the IAV-infected mice (median = 149, IQR = 137–155, n = 25) compared to the controls (median = 154, IQR = 144–160, n = 29); however, this was not significant (Mann–Whitney U test; p = 0.081; n = 54; Fig. 3A). Restraint stress induced a rise in blood glucose levels in all groups (Wilcoxon signed-rank test; all p < 0.001; Fig. 3B). The increase in glucose was calculated as delta (post—pre-stress) and visualized with contrasting results for both sexes. In females, the control group (median = 46.0, IQR = 35.5–63, n = 31) showed a greater rise (Mann–Whitney U test; p < 0.05; n = 53; Fig. 3B) than the IAV-infected mice (median = 33.5, IQR = 6.75–30.77, n = 22), whereas for males the results were inverse. The IAV-infected males (median = 46.0, IQR = 39–62, n = 25) had a higher rise (Mann–Whitney U test; p < 0.05; n = 54; Fig. 3B) than the control group (median = 37, IQR = 26–50, n = 29).

CORT was measured in the plasma collected at the beginning and end of the PND42 restraint stress for a randomly-selected subset of 7–11 animals per treatment and sex. In males, the pre-stress (baseline) levels of CORT were significantly higher in the control group (median = 52.2, IQR = 46.5–69.3, n = 7, Fig. 3C) than in the IAV-infected males (median = 24.8, IQR = 17.6–39.0, n = 11) prior to stress (Mann–Whitney U test; p < 0.001, test stat = 4.23; n = 18; Fig. 3B). This trend was not visible in females (Mann–Whitney U test; p = 0.8, n = 18; control: median = 42.61, IQR = 25.9–93, n = 9; IAV: median = 37.68, IQR = 30.4–49.4, n = 9; Fig. 3C). As expected, corticosterone levels rose significantly during the restraint stress (Wilcoxon signed rank test; all p < 0.05; Fig. 3D), with an overall stronger effect in females. However, there was no notable difference in the stress-induced rise in CORT levels (T1-T0) when comparing between the PND14 treatment groups in females (Fig. 3D). In males, we observed the expected stress-induced increase in CORT levels (Mann–Whitney U test; p = 0.069, n = 18; Fig. 3D) for both PND14 treatment groups, with a larger rise in CORT levels for mice infected with IAV at PND14 (median = 455, IQR = 390–554, n = 11) than in the control group (median = 345, IQR = 297–398, n = 7).

### Brain MRI

**Figure 4:**
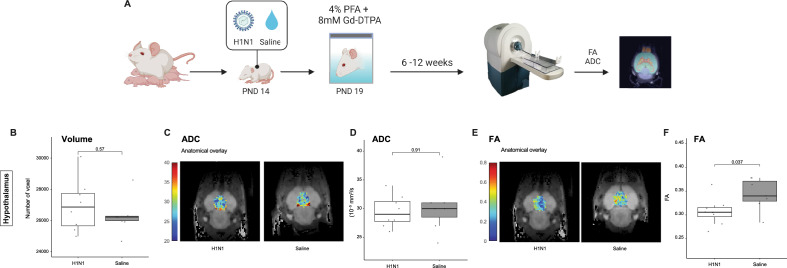
MRI schematic representation. A small group of mice at PND19 were sacrificed by decapitation and their entire heads were placed in tubes containing a cold solution of 4% Paraformaldehyde (PFA) and 8 mM Gadolium (Dotarem 0.5 mmol/ml, Guerbet). The tubes were then left at 4 °C for 6–12 weeks for the contrast agent to fully diffuse and stabilize in tissue prior to ex-vivo imaging. We carried out FSE3D and SE-DTI to obtain high-resolution anatomy images and maps of diffusion of water molecules within the brain. Boxplots of measurements and representative slice with ADC/FA overlay, focusing on the Hypothalamus region (above). Volume axes indicate dimensions in log10(voxels); ADCs are mapped in units of 10–5 mm2/s; FA units reach from 0 to 1.

Given the overwhelming literature on ELA in connection to structural changes in the brain, as well as behavioral and mental health outcomes^40–42^, we integrated neuroimaging data into our semi-holistic model. At PND19, we carried out FSE3D and SE-DTI to obtain high-resolution anatomy images and maps of diffusion of water molecules within the brain. We randomly picked 13 female animals from four litters (control = 6; H1N1 n = 7). Imaging was carried out ex vivo and using the mouse brain atlas (3D Brain Atlas, Invicro's VivoQuant™), 14 sub-regions were identified and mapped (Fig. 4 and Supplementary Data 2). Total volume (in voxels), apparent diffusion coefficient (ADC), and fractional anisotropy (FA) were assessed.

**Figure 5:**
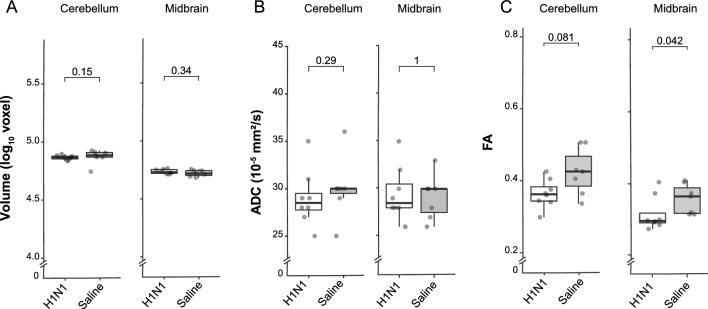
Brain MRI results; data were obtained from PND19. Boxplots of morphology and diffusion-related measurements. Volume axes indicate dimensions in log10(voxels); ADCs are mapped in units of 10–5 mm2/s; FA units reach from 0 to 1.

There were no significant differences in the volume of the different regions between treatments, in contrast to the significant differences in body weight measured at PND19. Furthermore, we did not detect significant differences in the ADC, the relative measure of the diffusion of water, between the treatments groups either. Interestingly, the FA values were overall lower in the IAV-infected mice, possibly reflecting a reduced connectivity within different brain regions for this group. These changes proved to be significant in the hypothalamus (Mann–Whitney U test; p < 0.05; Supplementary Data 2) and midbrain (Mann–Whitney U test; p < 0.05; Fig. 5), and also quite notable in the cerebellum, cortex and hippocampus (Mann–Whitney U test; all p ≥ 0.1; Fig. 5 and Supplemetary Data 2).

### Global gene expression analysis

Given the plethora of long-term non-immune effects of early-life infection, we compared gene expression in females between early-life treatments (control n = 5, IAV infected n = 5) in five different tissues. Liver and adrenal gland were chosen for their role in the altered glucose and corticosterone response to the restraint stressor which we applied, and the sub-cortical region, cerebellum and brainstem were chosen for the differences in FA. To ensure that these differences in gene expression were not due to immune cell infiltration into the selected tissues, we applied *consICA* method. Our deconvolution of the data set into 20 independent components resulted in only tissue-specific processes (e.g. xenobiotic metabolic process for liver, p.adj. = 7 × 10^−25^; regulation of synaptic plasticity for the cerebellum, p.adj. = 3 × 10^–8^) and did not detect any immune or immune-related signal (Supplementary Data 3). This suggests an absence of significant effects of the treatment on immune response, or residual differences in infiltrating immune cells in the considered organs.

**Figure 6:**
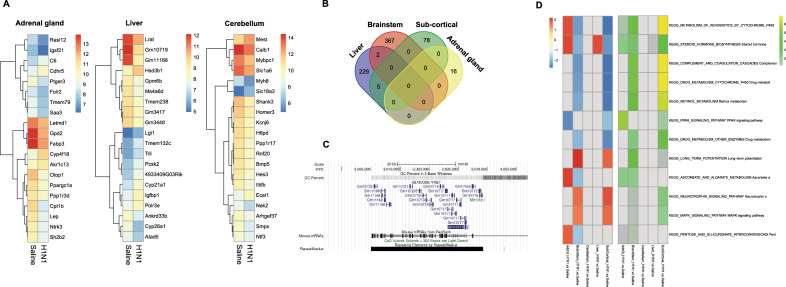
Differential expression analysis at PND60. (**A**) Heatmaps plotted for the top 20 differentially expressed genes (DEGs) between treatments for the adrenal gland, liver and subcortical region. Data represent the mean value of five independent animals. (**B**) Venn Diagram of DEG shared between different organs. (**C**) Genes clustered on chromosome 9, viewed in UCSC Genome Browser. (**D**) KEGG pathways activated in the tested tissues: left (red-blue) heatmap shows the normalized enrichment score (NES), with red representing values higher in H1N1 compared to Saline; right (green-yellow) heatmap highlights significance of the enrichment (−log10(p.adj.)).

To accurately report gene expression without using p-value cut-offs, we calculated an expression-specific score (using a combination of p.adj and log_2_ fold-change; see methods) and compared the top 20 scoring differentially expressed genes (DEGs) for every organ (3 displayed in Fig. 6A). Using this approach, we could not find any DEGs shared between different organs (Fig. 6B), so we then compared all genes with an expression specific score ≥ 0.5 for every organ in a Venn-Diagram. We found almost no DEGs shared between tissues, except 2 DEGs (*Nol3, G530012D18Rik*) shared between brainstem and liver, and 5 DEGs (*Gm10717, Gm10721, Gm10715, Gm11168, Gm17535*) shared between liver and sub-cortical region (Fig. 6B). Interestingly, all of the 5 DEGs shared between the liver and sub-cortical region belong to a gene tree family of repetitive elements clustered on chromosome 9 (Fig. 6C).

Given the paucity of DEGs seen at PND60 (Fig. 6D), we performed gene set enrichment analysis (GSEA) to test for significantly-enriched pathways and whether those are shared between tissues. Concentrating on the KEGG pathway databases, we found several pathways significantly impacted by early-life treatment and several of those were shared between tissues. The three most affected pathways were drug metabolism by cytochrome P450 (p.adj. = 1.06 × 10^–7^ for sub-cortical region and p.adj. = 1.41 × 10^–5^ for brainstem), complement and coagulation cascades (p.adj. = 1.06 × 10^–7^ for sub-cortical region and p.adj. = 1.41 × 10^–5^ for brainstem) and metabolism of xenobiotics by cytochrome 450 (p.adj. = 1.06 × 10^–7^ for sub-cortical region and p.adj. = 7.01 × 10^–5^ for brainstem). However, we found only one highly significant pathway for the cerebellum (Neuroactive ligand-receptor interaction, p.adj. = 5.26 × 10^–9^), which was not shared with any other tissue and is therefore not part of the heatmap (Fig. 6D).

## Discussion

Early life infections have been associated with a broad spectrum of later life effects, often associated with altered stress, endocrine or immune reactions. In this study, using a semi-holistic approach we were able to show long-term sexually dimorphic effects on multiple “non-immune” or “off-target” systems. A resolved early-life viral infection subsequently retarded the growth of males, increased baseline (pre-stress) blood glucose levels in females and lowered them in males, and reduced baseline corticosterone levels, although stress-induced changes remained unaffected. These changes were accompanied by significantly decreased in connectivity in the cerebellum, cortex and hippocampus, regions associated with the stress response, and specific gene expression signatures throughout the different tissues examined. This series of highly tissue-specific gene expression signatures that do not share common pathways provides clear confirmation of our previous human observational data, confirming that the ELA has independently affected each of the systems.

The first system we studied was the brain. ELA, in the form of psychosocial stress, has long been associated with aberrant neurodevelopmental patterns and eventually disease^43^, with recent studies identifying changes in behavior, emotion and attention, and reward-learning that were thought to be induced by changes in connectivity^44–46^. Here, we were able to show a significant reduction of connectivity in various parts of the brain, measured by fractional anisotropy after an ELI. We then used the MR imaging to select brain regions for gene expression profiling. Our microarray data identified two pathways in the brain: Nrf2, and cytochrome P450. The nuclear factor erythroid 2–related factor 2 (Nrf2) pathway regulates the cellular antioxidant defense^47^ in response to viral-replication induced oxidative stress while replicating^48–50^, and its’ long-term downregulation has been associated with chronic neurodegenerative diseases^51,52^. The downregulated Cytochrome P450 pathway enzymes are mainly active in liver metabolism, they have also been shown to be involved in brain micro-metabolism^53,54^. Interestingly, several cytochrome enzymes (e.g. *Cyp2d6, Cyp1a2, Cyp19, Cyp46*) are downregulated Parkinson’s disease [Reviewed in^55^] and inflammation of central nervous system tissues^53^. As we detected a similar downregulation of this pathway and reduced connectivity in movement-associated brain regions after our early-life respiratory infection, it is interesting to draw parallels with the similar movement disturbances and both Nrf and Cyp450 pathways seen in disorders such as dementia and Alzheimer’s disease^52,56,57^. Leading into the second system of interest, the hypothalamus, a key player of the HPA-axis, was also affected by viral infection both in our data and in previous reports^36,58^. As our model is a respiratory infection, this suggests a bi-directionality of the HPA-immune axis, with a peripheral IAV infection signaling to the brain^59–61^.

The HPA axis and adrenal steroid hormones such as GCs are essential regulators of the immune response suppressing inflammation. While lower adult GC levels and a blunted CORT response to stress have repeatedly been associated with early life stress^28,62^, a short term increase of GCs is tightly associated with viral infections and inflammation^63^. We hypothesized that hypercortisolemia would have a similar long-term effect early when induced by an early-life infection. We did not observe a decreased CORT stress response later in life, and baseline CORT levels were either equal or lower in ELI mice compared to control. However, an excess of GC leads to immunosuppression, fat accumulation, insulin resistance, hypertension and depression^64^ that concords with the general ELA phenotype^37,65^. This agrees with our findings of baseline higher blood glucose levels and higher activity of steroidogenesis pathways in the liver and adrenal gland in females after ELI. While acute IAV infection has already been associated with a dysregulation in the host glucose metabolism^66–69^, recent studies have identified a direct link between IAV infection and the insulin signaling pathway^70^, and the differentiation of white and brown adipocytes^71^, leading to long-term changes in energy metabolism. Similar to Ayari et al., we also found that several pathways associated with energy metabolism and oxidative stress were altered, several weeks after infection. Furthermore, we saw an increase in the activity of the insulin signaling pathway, as well as decreased PPAR signaling, both involved in lipid and glucose metabolism, and neuro-inflammation^72–74^. We have recently shown that there is ample evidence that ELA can result in perturbed energy metabolism and hypothesized that it can fundamentally alter gluconeogenesis and therefore increase the risk of developing metabolic disorders^75^. As such, we were particularly interested in the stress-induced glucose rise. This is a secondary readout from the stress-system that may either be under control of the HPA axis hormones, or under under vagal control^75–79^. This has been one previous report that neurons within the solitary tract and dorsal motor nucleus of the vagal nerve are able to actively decrease hepatic gluconeogenesis, as well as modulate the expression of gluconeogenic enzymes, and glucose production^77^. However, we were not able to identify any changes in these brain regions in our MRI connectivity data and expression data was not collected here.

Our study has several limitations. We chose a mouse-adapted influenza virus because it is cleared primarily by the innate immune system. At PND14 the mouse adaptive immune system, required for clearing the majority of viruses is not fully developed. As such, we can probably extrapolate our results to the naïve human immune system in the early post-natal period, however, extrapolation to other viral infections such as CMV generally occurring later in life will be difficult. Similarly, our simplified infection model of ELA has only one element of the multipartite human ELA exposure and does not represent the full extent of the human clinical situation^80–82^. Nevertheless, simplifying the exposure to a viral infection that was cleared by the innate immune system allowed us to clearly show that many tissues were transcriptionally programmed independently. Furthermore, we were able to clearly state that there was no residual signature of immune infiltration or signalling after the infection was cleared. This agrees with undetectable levels of IL-2, -6, -10 and IFNγ in the BALF, although residual very low levels of TNFα around the lower limit of detection were observed. The results from this simplified early-life exposure model are in line with our recent “stem cell” hypothesis^39^. The challenge is now to combine this ELI with other elements of the three-hit DOHaD model, incorporating for example, genetic and later-life factors, together with the isolation of the stem cells in the tissues that we have shown here to be transcriptionally programmed. Importantly, stem cells have been identified in all the transcriptionally programmed tissues here, providing a sound basis for future studies^39^.

In summary, this is one of the very few studies investigating the long-term effects of ELA using a non-psychosocial stimulus. At the same time, we used a semi-holistic approach, “following” the phenotypic and metabolic changes seen throughout different tissues applying state-of-the-art techniques. Early life IAV infection leads to a distinguishable phenotype (lighter in weight, higher in blood glucose), reduced brain connectivity in areas responsible for memory, motor function and hormonal control, as well as a dysregulation of gene pathways responsible for steroid and energy metabolism shared between different tissues (liver, adrenal gland and brain) . Additionally, we have demonstrated a clear sex difference in pathology and phenotype that mirrors outcomes seen in human cohorts. The observed sexual dimorphism will hopefully fuel further investigation into this field and inspire future ELA research to include both sexes into their studies.

## Materials and methods

### Husbandry conditions and breeding of mice

BALB/cJRj mice were initially obtained from Janvier Labs (Genest-Saint-Isle, France) and subsequently bred in-house. All animals were maintained in ventilated cages (Tecniplast, Buguggiate, Italy) with standard rodent diet (SDS Diets, Witham, UK) and drinking water ad libitum. Enrichment was offered in the form of sterile cardboard tunnels, mouse houses (Tecniplast) and paper nesting material. For the experimental breeding, in-house bred mice (F0, age 10–20 weeks) were randomly allocated into breeding trios/pairs to form experimental groups. One week before delivery, the pregnant dams were placed in individually-ventilated ISOcages (Tecniplast) and given additional nesting material and cardboard tunnels. The number of pups (F1) was determined on post-natal day 2/3 and litter size was reduced to a maximum of 8 pups/litter when necessary. The pups were ear-marked on PND12. We exposed 24 litters of pups (12 litters for each condition H1N1 or PBS) to our early-life treatment (post-natal day 14; PND14) of either influenza-A-virus (IAV) dissolved in Phosphate-buffered saline (PBS) or only PBS as control (Fig. 1A). At PND56 animals were re-exposed to PBS except for a small group (Supplementary Data 1) that were re-exposed to IAV to serve as positive controls for IAV infection at sacrifice. All experimental procedures were performed following the approval of the Animal Welfare Structure of the Luxembourg Institute of Health (#DII-2017–15), in accordance with European Union directive 2010/63/EU as well as local guidelines, and reported according to the ARRIVE guidelines (https://arriveguidelines.org).

### Virus expansion, TCID50 and LD50 determination

For all infections, we used the H1N1 influenza virus strain A/Puerto Rico/8/34 (obtained from Pr. C.P Muller; WHO Collaborative Centre Luxembourg). Virus stocks were expanded on Madin-Darby canine kidney (MDCK) cells and the half tissue culture infectious dose (TCID50) was determined by plaque assay on MDCK cells as described previously^83^. After 72h, the cytopathic effect per-well was determined by two observers; TCID50 and focus forming units (FFU) were calculated using a method provided by Dr. Marco Binder described in^84^. Virus aliquots were frozen in liquid nitrogen and only thawed once.

For the determination of the half lethal dose (LD50), 7–10-week-old mice were lightly anaesthetized by inhaling Isoflurane (1mL/mL; cp-pharma, Burgdorf, Germany) and 50 µL of virus suspension (10^3^ – 10^5^ FFU/mL in PBS) was given by intranasal way. The animals were scored daily for weight and signs of infection over 16 days as described by Koch and colleagues with minor modifications^85^ (Supplementary Data 4 and 5) and were euthanized if necessary. LD50 was calculated as 10^3^^77^ FFU/mL, and 10^3^^8^ FFU/mL respectively from the morbidity and mortality scores, using the methods of Reed & Muench and Ramakrishnan respectively^86,87^. The mean value 6 × 10^3^ FFU/mL was subsequently used in all experiments.

### PND14 infection and PND56 challenge

At PND14 juvenile F1 mice were administered PBS (Control group; Lonza, Basel, Switzerland) or influenza A virus (IAV group). For intranasal (i.n.) inoculation, the mice were lightly anaesthetized by inhaling Isoflurane (cp-pharma), and 25 µL virus suspension (1500 FFU IAV in PBS) or PBS were administered through droplets into the nose of the animals which were subsequently scored and weighed daily for 14 days. After weaning, the mice were kept in same-sex and same-treatment cages to avoid cross-contamination. On PND56 animals underwent a hetero- or homotypical challenge with either PBS or IAV and subsequently scored until sacrifice (Supplementary Data 4). The animals were sacrificed on PND60/61 by cervical dislocation. Immeadiatly after sacrifice, whole blood was collected by cardiac puncture as well as bronchoalveolar lavage (BAL) fluid by inserting a flexible tube through a cut in the trachea and washing the lungs with 700μL PBS. Following this complete brains, thymus, lungs, adrenal glands, and the distal tip of the left lobe of the liver were harvested and stored at –80°C before analysis.

### Restraint stress

On PND42 (± 1), we performed a restraint stress test by placing the animals in a transparent tube (according to the size of the animal: Ø2.86cm; BioSeb, Vitrolles, France or Ø2.48cm; G&P Kunsstofftechnik, Kassel, Germany) for 60 min. Blood (approx. 50µL) was collected (in EDTA treated capillary tubes; Sarstedt, Nümbrecht, Germany) by incision of a lateral tail vein, immediately after placing and approximately 1 min before releasing the animal from the restrainer. Plasma was subsequently separated by one-step centrifugation (2000 × *g*, 10 min, 4 °C) and stored at − 80 °C until analysis. Glucose measurements were performed immediately using a standard blood glucose meter (Accu-Check Aviva; Roche, Basel, Switzerland).

Corticosterone competitive ELISA.

Plasma corticosterone levels were measured using Corticosterone High Sensitivity EIA kit (Immunodiagnosticsystems) according to the manufacturer’s instructions. Samples were diluted in assay diluent, either 50-fold (baseline samples pre-stress) or 100-fold (one hour of restraint).

### Viral qPCR detection

For each mouse, one complete lung lobe was homogenized with a TissueLyzer II (Qiagen) and total RNA was extracted using Qiagen AllPrep DNA/RNA/miRNA kit according to the manufacturer’s instructions. Qiagen One Step kit was used for reverse transcription and H1N1 qPCR detection was performed as previously described^88^. As a positive control, we used PND56 IAV infected animals sacrificed at PND60 without early-life treatment.

### Magnetic resonance imaging

A small group of mice were anesthetized at PND19 using 4% isofluorane in air and sacrificed by decapitation. The entire heads were placed in tubes containing a cold solution of 4% Paraformaldehyde (PFA) and 8 mM Gadolium (Dotarem 0.5 mmol/ml, Guerbet). The tubes were then left at 4°C for 6–12 weeks for the contrast agent to fully diffuse and stabilize in tissue prior to ex-vivo imaging.

Magnetic Resonance Imaging (MRI) was performed on a 3T preclinical horizontal bore scanner (MR Solutions, Guilford, UK), equipped with a quadrature volume coil designed for rodent head imaging, with a 17 cm horizontal bore. On analysis day, the heads were placed in a custom-made 3D holder filled with fluorinert (3M, MN, USA) and anatomical and structural MRI sequences were acquired. Three dimensional Fast Spin Echo (FSE3D) anatomical series were used to calculate the volumes of brain sub-regions. Spin Echo Diffusion Tensor Imaging (SE-DTI) was used to calculate the diffusions parameters as described below. The details of each sequence have been reported in the Supplementary Data 6. The 3D Brain Atlas (Invicro's VivoQuant™) provided 14 sub-regions and the analysis of the changes in brain volume or in the diffusion parameters was performed with Matlab 2020b (MathWorks, MA, USA). The atlas provided 14 sub-regions and the analysis was completed for all sub-regions.

### Gene expression

The complete brains were taken from the skull within 10 min after sacrifice, snap-frozen in -80°C cold isopentane (VWR, #24872.260) and stored at – 80 °C until dissection. For brain dissection, the whole brains were free-hand cut by a trained operator at approximately – 25 °C and cerebellum, brain stem (medulla + pons), and the sub-cortical region (comprised of hippocampus, amygdala and parts of the cerebral cortex grey matter) were extracted and subsequently homogenized using a TissueLyzer II (Qiagen) and complete RNA was extracted using Qiagen AllPrep DNA/RNA/miRNA kit according to the manufacturer’s instructions for fatty tissues.

The whole thymus, both adrenal glands and a piece of the distal tip of left liver lope were cut out within 20 min after sacrifice and stored in RNA later solution (invitrogen) at -80°C until they were homogenized using a TissueLyzer II (Qiagen) and complete RNA was extracted using Qiagen AllPrep DNA/RNA/miRNA kit according to the manufacturer’s instructions.

RNA expression analysis was performed with Affymetrix Mouse Clariom S® microarray using 3´IVT Pico Kit (Thermo Fisher Scientific) according to the manufacturer’s instructions. In brief, 10 ng of total RNA was reverse transcribed, from both poly-A tails at random, to capture coding and non-coding forms of RNA. Complementary RNA (cRNA) amplification was achieved using low-cycle PCR followed by linear amplification using T7 *in-vitro* transcription. The cRNA was then converted into biotinylated double-stranded cDNA (ds-cDNA) hybridization targets for unbiased coverage of the transcriptome. Ds-cDNA (3 μg) was injected into the array cartridge and hybridized for 16 h at 45°C at 60 rpm. The arrays were subsequently washed, stained (*GeneChip™* Fluidic Station 450*)* and scanned (*GeneChip™* Scanner 3000).

### Data analysis

Unless otherwise stated, statistical tests were done in R (4.0.2) using R Studio interface. Overall, we used the following packages to load, clean, filter, manipulate, and visualize data: *tidyverse* (v1.3.1), *ggpubr* (v0.7.0), *broom* (v0.7.6), *ggstatsplot* (v0.7.1).

#### Mice phenotype

We used a two-way analysis of covariance (ANCOVA) to evaluate the effect of grouping variables (sex and PND14 treatment) on our outcome variable (body weight), after adjusting for the covariate (time), using the *rstatix* (v0.7.0) package. For post-hoc analysis, pairwise comparisons of estimated marginal means (emmeans) with Bonferroni correction were performed for all significant variables of the ANCOVA. The data was filtered by sex, and pairwise Mann–Whitney U/Wilcoxon tests were used on body weight for individual time points (post-natal days) and visualized using the *ggpubr* package.

#### Diffusion MRI

NordicICE (NordicNeuroLab, Bergen, Norway) was used to analyze diffusion related images. ADC maps (scaling of units: 10^–5^ mm^2^/s) were computed using the equation $S={S}_{0}{e}^{(-b*ADC)}$, where ‘S’ and ‘S0’ indicate the diffusion signals recorded in the presence and absence of diffusion (b = 0) respectively. The direction of diffusion within a 3D voxel is reflected by the FA maps and characterised by eigenvalues (λ) in multiple directions. The equation used to calculate FA maps is: $FA= \sqrt{\frac{{({\uplambda }_{1}-{\uplambda }_{2})}^{2}+{({\uplambda }_{2}-{\uplambda }_{2})}^{2}+{({\uplambda }_{1}-{\uplambda }_{3})}^{2}}{2({\lambda }_{1}^{2}+{\lambda }_{2}^{2}+{\lambda }_{3}^{2})}}$. The FA values can vary between 1 and 0. Matlab was used to calculate volumes and quantify diffusion parameters in the different brain regions after registration of the MR images to a mouse brain atlas. Central tendency reported for the regions of interest (ROIs) are median values. FA and ADC median values were exported into R Studio and Mann–Whitney U/Wilcoxon tests were performed for each brain region.

#### Microarrays

CEL files were processed (normalized and summarized) using ThermoFisher Transcriptome Analysis Console (TAC, v.4.0.1.36) by SST-RMA method that corrects bias related to GC-content. Probesets were then summarized to gene level. Further analysis was performed by R/Bioconductor (v.4.0.0). In short, in order to improve sensitivity, we focused on expressed genes, using a soft threshold as described in^89^ (genes showing log_2_ expression > 8 in at least one sample were preserved for further statistical analysis), resulting in 14787 genes. Differential expression analysis was performed using *limma* package (v.3.44.3)^90^. A global linear two-factor model was built for the entire dataset, followed by analysis of contrasts. Benjamini-Hochberg’s FDR adjustment was applied to p-values for multiple hypotheses correction. In order to report a limited number of differentially expressed genes, the following score was calculated as a combination of adjusted p-value and a log_2_ fold-change measure, similar to^91^:

score = –log_10_(p.adj.)·|log_2_FC|. The top 20 genes were presented on row-scaled heatmaps (package *pheatmap*, v.1.0.12).

Taking into account that each sample represented an ensemble of cell types, we performed deconvolution using a consensus independent component analysis (consICA) method^92^. This data-driven method characterizes the activity of biological processes and gives estimation of cell types.

Gene set enrichment analysis (GSEA) was performed by clusterProfiler package (v.3.16.1)^93^ using gene sets defined be *msigdbr* package (v.7.2.1) within collections (WIKIPATHWAY, KEGG Pathway^94^). For significantly enriched gene sets, we reported p.adj. showing statistical significance, and a normalized enrichment score (NES) representing the direction of change. As a non-parametric approach to data analysis, we considered samples in compared groups and calculated AUC values for the genes using *caTools* package (v.1.18.1). The top candidate genes were then reported.

### Supplementary Information

Supplementary Information.

## Data Availability

All data and analytical code from this study are available upon reasonable request to J.D.T.
