# Supplementary material for: Early-life influenza A (H1N1) infection independently programs brain connectivity, HPA AXIS and tissue-specific gene expression profiles
Source: Sci Rep. 2024 Mar 11;14:5898. doi: 10.1038/s41598-024-56601-5 (PMC10928197; doi:10.1038/s41598-024-56601-5)
Supplement: Supplementary file 1 — Supplementary Information. [file 41598_2024_56601_MOESM1_ESM.pdf]

Early-life Influenza A (H1N1) infection independently programs brain connectivity, HPA axis and tissue-specific gene expression profiles

Myriam P. Merz[1,2,†], Snehaa V. Seal[1,2], Nathalie Grova[1,3], Sophie Meriaux[1], Pauline Guebels[1], Georgia Kanli[4,5,], Elise Mommaerts[6], Nathalie Nicot[6], Tony Kaoma[7], Olivier Keunen[4,5], Petr V. Nazarov[7,8], Jonathan D. Turner[1,*]

[1] Immune Endocrine and Epigenetics Research group, Department of Infection and Immunity, Luxembourg Institute of Health (LIH), 29 rue Henri Koch, 4354 Esch-sur-Alzette, Luxembourg

[2] Faculty of Science, Technology and Medicine, University of Luxembourg, 2 avenue de Université, L-4365 Esch-sur-Alzette, Luxembourg

[3] Université de Lorraine, Inserm U1256, NGERE, Nutrition-Génétique et Exposition aux Risques Environnementaux - 54000 Nancy, France

[4] In vivo Imaging Platform, Luxembourg Institute of Health, 1445 Strassen, Luxembourg

[5] Translational Radiomics, Department of Cancer Research, Luxembourg Institute of Health, 1526 Luxembourg, Luxembourg

[6] LuxGen Genome Center, Luxembourg Institute of Health, Laboratoire National de Santé 3555 Dudelange, Luxembourg

[7] Bioinformatics Platform, Data Integration and Analysis Unit, Luxembourg Institute of Health, 1445 Strassen, Luxembourg

[8] Multiomics Data Science Research group, Department of Cancer Research, Luxembourg Institute of Health, 1445 Strassen, Luxembourg

Supplementary Data

Supplementary Data 1: PCR quantification of IAV (H1N1) RNA extracted from homogenised mouse lungs was reverse transcribed using SuperScript 3 and H1N1 was amplified using the protocol of Spackman et al (Reference 89). H1N1 was not detected in any of the animals an PND 56. Ct values are given, and NA represents samples for which no amplification was observed.

mouse_ID	Ct	PCR_plate	note	DOB	generation	PND14_date	PND14_treatment	PND56_date	PND56_treatment	sex
5147	**20.36**	1	posControl	20/09/2019	F1	04/10/2019	Sal	21/11/2019	H1N1	f
3009	**23.94**	2	posControl	11/09/2018	F1	25/09/2018	Sal	06/11/2018	H1N1	m
3011	**34.28**	2	posControl	11/09/2018	F1	25/09/2018	Sal	06/11/2018	H1N1	f
3007	**35.52**	3	posControl	11/09/2018	F1	25/09/2018	Sal	06/11/2018	H1N1	m
5152	**NA**	2	posControl	20/09/2019	F1	04/10/2019	Sal	21/11/2019	H1N1	m
3001	**NA**	1	NA	05/09/2018	F1	19/09/2018	Sal	31/10/2018	Sal	m
4042	**NA**	1	NA	25/02/2019	F1	11/03/2019	Sal	22/04/2019	Sal	f
4048	**NA**	3	NA	26/02/2019	F1	12/03/2019	Sal	23/04/2019	Sal	m
5184	**NA**	2	NA	26/10/2019	F1	09/11/2019	Sal	14/12/2019	Sal	m
6036	**NA**	1	NA	11/01/2020	F1	25/01/2020	Sal	07/03/2020	Sal	f
6039	**NA**	3	NA	11/01/2020	F1	25/01/2020	Sal	07/03/2020	Sal	f
6062	**NA**	2	NA	16/02/2020	F1	02/03/2020	H1N1	12/04/2020	Sal	f
3013	**NA**	1	NA	14/09/2018	F1	28/09/2018	H1N1	09/11/2018	Sal	f
3015	**NA**	1	NA	14/09/2018	F1	28/09/2018	H1N1	09/11/2018	Sal	m
4002	**NA**	1	NA	09/02/2019	F1	23/02/2019	H1N1	05/04/2019	Sal	m
4007	**NA**	1	NA	12/02/2019	F1	26/02/2019	H1N1	11/04/2019	Sal	f

Data 3: Microarray Immune infiltration

Deconvolution: In order to check for the presence of specific cellular populations and processes, we applied consensus independent component analysis (consICA) (Nazarov et al., 2019). It was already shown that the method successfully identifies variable cell subtypes and cellular processes in bulk-sample transcriptomics data, especially linked to immune and proliferative cells (Nazarov et al., 2019, Golebiewska et al., 2020). The method was applied as described in (Nazarov et al., 2019) directly on the dataset, thus avoiding using of a reference data. Number of independent components was fixed to 20.

Results: Our deconvolution of the data set result in tissue-specific processes (e.g. xenobiotic metabolic process for liver, FDR=1e−22, regulation of synaptic plasticity, FDR=9e-8, for cerebellum) but did not detect any immune signal. This suggests no significant effect of the treatment on immune response in the considered organs.

TISSUE	PROCESS	FDR
sub-cortical	chemical synaptic transmission	2e−27
cerebellum	regulation of synaptic plasticity	3e−08
sub-cortical, cerebellum, brain stem	chemical synaptic transmission	2e−27
liver	xenobiotic metabolic process	7e−25
liver	exogenous drug catabolic process	2e−24
adrenal gland	fatty acid metabolic process	6e−14
adrenal gland	steroid hormone biosynthetic process	5e−03

Data 4: Scoring system for IAV sickness symptoms. Template from (Koch et al., 2016) with modifications.

Feature	Symptoms	points	Day1	Day2	Day3	Day ...
Appearance	Normal (smooth coat, clear eyes/nose)	0				
	Hunched and/or fluffed	1				
	Ocular discharge and/or edema	3				
	Pale, white mucus membranes/skin	6				
	Blue mucus membranes/skin (cyanosis	12				
Respiratory rate	Normal breathing	0				
	Increased breathing (*subjective* recognition by experimenter)	3				
	Increased breathing (*obvious*; double normal rate, rapid, shallow)	6				
	Abdominal breathing (+/-gasping or open mouth breathing)	12				
General behavior	Normal (based in baseline observations)	0				
	Decreased mobility	2				
	Ataxia, wobbly, weak	6				
	Inability to stand	12				
Provoked behavior	Normal (moves when cage is disturbed, runs from hand)	0				
	Subdued; responds to stimulation (moves away briskly)	1				
	Subdued even to stimulation (moves away slowly)	3				
	Unresponsive to gentle prodding	6				
	Does not right when placed on side within 5 seconds	12				
		**Weight (in grams):**				
		**% loss (compared to day 1):**				
Weight loss	< 10%	0				
	10-15%	2				
	15-20%	6				
	20-25%	10				
	≥25%	12				
		**Total Score:**				
		**Notes:**				
		**Initials of experimenter/caretaker:**				

**Score**	
< 6	Normal
6–11	Morbid: Monitor at least 3 times per day; notify appropriate personnel immediately
≥ 12	Moribund: Notify responsible personnel immediately<br>If any single criterion is 12 or higher = euthanize immediately

Data 5:LD50 morbidity and mortality

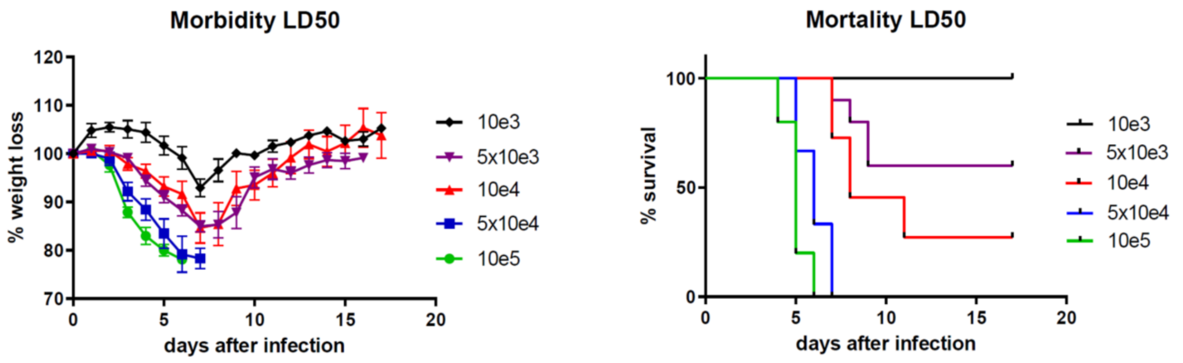

Data 6. MRI scan protocol. SR: Spatial Resolution, Sl: Number of Slices, TE: Echo Time, TR: Repetition Time, ET: Echo Train, AVG: Number of Averages, SD: Scan Duration, NOB: number of b-value, NOD: number of diffusion direction.

SEQUENCE	PURPOSE	PARAMETERS
**SE-DTI**	Mapping of Franctional Anisotropy (FA)/ preferential direction of diffusion	**SR:** 0.16 × 0.16 × 1 mm; **Sl:** 20; **TE\TR:** 22\800 ms; **AVG:** 5; **NOB:** 1; **b-value:** 1000; **NOD:** 7; **SD:** 49min44sec
**µMRI - FSE**	High resolution anatomy	**SR:** 0.08 × 0.08 × 0.2 mm ; **Sl:** 64 ; **TE\TR:** 17\3000 ms; **ET:** 8; **AVG:** 2; **SD:** 3h31min42sec
